# Cerebral Schistosomiasis in the Napu Valley, Central Sulawesi, Indonesia

**DOI:** 10.1155/carm/4781807

**Published:** 2025-07-01

**Authors:** Prawesty Diah Utami, Yunita Surya Pratiwi, Retno Budiarti, Wienta Diarsvitri

**Affiliations:** ^1^Parasitology Department, Faculty of Medicine, Hang Tuah University, Surabaya, East Java, Indonesia; ^2^Maholo Community Health Center, Poso, Central Sulawesi, Indonesia; ^3^Microbiology Department, Faculty of Medicine, Hang Tuah University, Surabaya, East Java, Indonesia; ^4^Department of Community Medicine, Faculty of Medicine, Hang Tuah University, Surabaya, East Java, Indonesia

**Keywords:** cerebral, neurology manifestation, *S. japonicum*, schistosomiasis

## Abstract

Schistosomiasis is one of the neglected tropical diseases caused by parasitic worm infections of the genus *Schistosoma*. Involvement of the brain in schistosomiasis represents a particularly severe manifestation of the infection. Accurate diagnosis and appropriate treatment of cerebral schistosomiasis are essential, especially in our healthcare facility located in a remote area of Indonesia, where available resources are highly limited. We reported a 31-year-old female patient complaining of tonic-clonic convulsions. Before experiencing seizures, the patient reported experiencing headaches for 6 months. The patient's anamnesis regarding her social life revealed that she has been employed on a plantation for 15 years; the plantation serves as a natural habitat for snails, intermediate hosts for *Schistosoma* sp. Serological examinations were not performed due to the constraints of diagnostic instruments in the region. Cerebral schistosomiasis diagnosis was verified based on biopsy, stool examination, and CT scan results. She was admitted with a combination of steroids and praziquantel at a dosage of 60 mg/kg single dose. She was released after 14 days in satisfactory overall health. The follow-up CT scan revealed improvement, corroborated by the patient's clinical recovery. This report emphasizes the diagnostic obstacles associated with cerebral schistosomiasis, particularly in remote regions and resource-limited settings in Indonesia. Despite the absence of serological testing, a definitive diagnosis was successfully established through radiological imaging, stool microscopic examination, and brain tissue biopsy (histopathological analysis) which revealed *Schistosoma* eggs surrounded by granulomatous inflammation. The patient presented with space-occupying brain lesions and neurological symptoms, but without hepatic involvement, making the diagnosis less straightforward. This case illustrates the significance of recognizing cerebral schistosomiasis as a differential diagnosis in patients presenting cerebral lesions in endemic locations. Diagnosis of cerebral schistosomiasis based on a detailed social occupational history correlated with radiological imaging, stool microscopic examination, and brain tissue biopsy (histopathological analysis) is essential when other diagnostic tools (serological testing) are unavailable.

## 1. Introduction

Schistosomiasis is classified as a neglected tropical disease that arises from infections with *Schistosoma* species, parasitic worms, which consist of three species that often infect humans: *S. haematobium, S. mansoni*, and *S. japonicum* [1, 2]. These worms live in freshwater; the infection generally begins when the sufferer comes into contact with contaminated water while swimming, washing, or paddling a boat [3]. Parasitic worms or cercaria enter the body through the skin, lodge in the body, and then mature after 40 days [4]. Schistosomiasis primarily impacts low- and middle-income nations located within tropical and subtropical zones, resulting in an annual infection rate of around 250 million individuals [5]. The World Health Organization (WHO) has documented the presence of schistosomiasis in 78 nations, characterized by 51 requiring the administration of preventive drugs due to the high transmission level [6]. In 2016, schistosomiasis contributed to a global disease burden of 2.5 million Disability-Adjusted Life Years (DALYs) and was responsible for about 24,000 mortalities [7]. The presented data highlight the serious public health implications of schistosomiasis, with its elimination constituting a central aim of the 2021–2030 Neglected Tropical Diseases (NTD) roadmap [8].

Schistosomiasis is widespread in tropical places globally and considered the second leading parasitic disease in terms of its socioeconomic burden, following malaria [9]. The socioeconomic impact of schistosomiasis is substantial, as it often affects the productivity of individuals due to the chronic nature of the disease and the high treatment costs [10].

In Africa and Asia, schistosomiasis remains a prominent issue affecting public health [11]. *S. japonicum* is the most common species in Asia, with high prevalence in China, Philippines, and Indonesia [12]. In Indonesia, Schistosomiasis is found in Central Sulawesi Province, especially in Poso and Sigi Regencies [13]. This worm is endemic in 28 villages in Central Sulawesi, especially in Poso Regency (Bada and Napu highlands) and Sigi Regency (Lindu highlands) [14]. Indonesia is the last country in the Southeast Asian region that is still trying to eliminate schistosomiasis [15].

Each species of *Schistosoma*'s adult worms has predilection sites for infection. *S. japonicum* and *S. mansoni* demonstrate a tropism for the mesenteric venules of the intestinal tract, whereas *S. haematobium* preferentially colonizes the bladder's venous plexus. Within these specific host venous plexuses, adult worms establish mating pairs and engage in sexual reproduction for the duration of their 3- to 5-year existence, producing eggs expelled in either feces or urine [16, 17]. Urogenital involvement includes the occurrence of blood in the urine and fibrotic changes within the bladder tissue, injury to the kidneys, and a heightened susceptibility to malignancies of the bladder [18]. Gastrointestinal manifestations potentially present as abdominal discomfort, occasionally accompanied by diarrhea and hematochezia [19]. In uncommon instances, Schistosomiasis can result in central nervous system (CNS) symptoms due to the inflammatory response triggered by the deposition of eggs [20]. Among the various clinical manifestations, cerebral schistosomiasis is recognized as one of the most severe. The most frequent cause of cerebral schistosomiasis is *S. japonicum*, while numerous cases are attributed to *S. mansoni* documented in the literature [21].

A previous study documented cases of cerebral schistosomiasis caused by *Schistosoma haematobium*, with seven patients diagnosed through the microscopic examination revealing within urine or stool samples, and fifteen cases identified via immunological assays. In these instances, the cerebral involvement was attributed to the migration of eggs from distant body sites, rather than the presence of adult worms in the brain tissue itself, as no adult parasites were found in the examined brain specimens. Moreover, there were no indications of portal hypertension or liver cirrhosis—conditions that might facilitate the movement of worms affecting the CNS. The occurrence of egg deposits may be the result of either abnormal worm migration or embolic transport of eggs from another site [22]. In a separate report, three Filipino male patients were diagnosed with cerebral schistosomiasis linked to *S. mansoni*. This species is often overlooked as a significant contributor to cerebral schistosomiasis. Among all *Schistosoma* species, *S. mansoni* is most commonly associated with spinal cord pathology. Cerebral schistosomiasis arises when schistosome eggs fail to exit the body via the urine or feces and instead migrate to the CNS, where they release proteolytic enzymes. These enzymes provoke eosinophilic inflammation and granuloma formation, which can subsequently be replaced by fibrotic tissue [23].

Praziquantel (PZQ) is the first-line treatment for schistosomiasis, including cerebral schistosomiasis, and is effective against all *Schistosoma* species. However, it relies on a competent host immune response to maximize efficacy. PZQ primarily targets mature worms, with limited activity against juvenile forms, making it less effective during the early stages of infection. In cerebral schistosomiasis, especially the acute form, monotherapy with PZQ could prove insufficient and has been reported to cause severe hypersensitivity reactions and even clinical deterioration. Consequently, corticosteroids are often coadministered to control inflammation and reduce eosinophil- and toxin-mediated neurological damage. In some cases, delaying PZQ administration until after neurological stabilization is recommended. Additionally, PZQ may cause adverse effects such as abdominal discomfort, headache, and dizziness. These limitations highlight the need for cautious use and combined therapy in managing cerebral schistosomiasis [23, 24].

Accurate diagnosis and appropriate treatment of cerebral schistosomiasis are essential, especially in our healthcare facility located in a remote area of Indonesia, where available resources are highly limited. The diagnosis and management of cerebral schistosomiasis posed a significant challenge in this case. This case report did not utilize serological testing due to the limited availability of diagnostic facilities in the referral hospital. Therefore, the diagnosis was primarily established based on radiological imaging, stool microscopic examination, and brain tissue biopsy (histopathological analysis). This report describes an active cerebral schistosomiasis case in a woman who presented clinically with headache and convulsive episodes. Computed tomography scanning revealed a lesion that occupied the left parieto-occipital area with perifocal edema. This is an uncommon instance of the disease. This case was reported to raise awareness of this rare neurological manifestation of schistosomiasis, particularly in a resource-limited healthcare setting.

## 2. Case Report

A previously healthy female patient, who was 31 years old, presented herself to our community health center outpatient clinic complaining of tonic–clonic convulsions. Before experiencing seizures, the patient reported experiencing headaches for 6 months. The patient sought the advice of a midwife and was administered an analgesic; however, the symptoms were not alleviated. Subsequently, the patient had four tonic–clonic seizures, which necessitated a visit to the community health center. The patient was directly referred to the referral hospital for additional evaluation. This patient was first suspected glioma. The patient's anamnesis regarding her social life revealed that she has been employed on a plantation for 15 years; the plantation serves as a natural habitat for snails, intermediate hosts for *Schistosoma* sp.

Physical examination revealed the absence of additional related symptoms, yet a detailed clinical assessment demonstrated that the patient's health was markedly poor. However, no signs of paleness, jaundice of the skin or eyes, or bluish discoloration were noted. All of his vital indicators were within the usual range. During the neurological assessment, the patient was conscious and aware of their surroundings, including the current time, location, and identity. The doctor scored a perfect 15 out of 15 on the Glasgow Coma Scale, indicating normal brain function. Additionally, their pupils reacted normally and were of equal size on both sides. All joints exhibited normal muscular tone and reflex activity, and the patient exhibited motoric strength Grade 5+, affecting the right-sided upper and lower extremities. The systemic evaluation was within normal limits.

**Figure 1:**
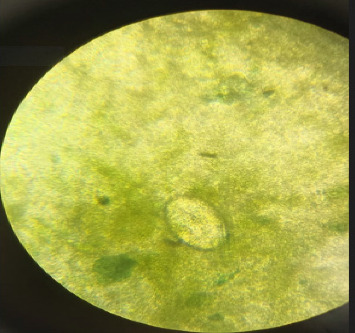
Microscopic stool examination: *S. japonicum*'s egg.

**Figure 2:**
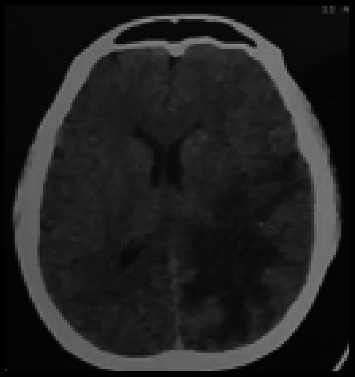
Computed tomography of the brain revealed a hypodense lesion in the left parieto-occipital region accompanied by marked perifocal edema.

Table 1 above shows an elevation in both eosinophils and procalcitonin, indicating immune activation and systemic inflammation, which is often associated with infections. Then, microscopic examination of the stool sample was performed using the direct smear technique. Stool examination revealed eggs of *S. japonicum* (Figure 1). Thorax photos showed no abnormality. The neurosurgeon did a CT scan based on the patient's anamnesis, a physical examination, and primary laboratory results (Figure 2). CT scan revealed a hypodense area on the left parieto-occipital area with perifocal edema. Analysis of cerebral spinal fluid was not implemented.

Imaging via computed tomography showed a lesion localized in the left parieto-occipital region with adjacent perifocal edema. Based on the CT scan result, the doctor hypothesizes that the patient is experiencing gliosis on the left parieto-occipital region. The doctor accomplished a partial craniotomy to eliminate a cerebral mass and establish a diagnosis. Brain tissue biopsy was performed for the diagnosis of cerebral schistosomiasis. The histopathological analysis of the substance revealed gliosis with the following findings: Neural parenchyma with embedded *Schistosoma* eggs, encased within granulomatous inflammatory cells. A perivascular cuff of lymphocytes was observed. Serological examinations were not feasible due to the constraints of diagnostic instruments in the referral hospital in our region. CT scan, brain tissue biopsy (histopathological analysis), and stool examination using direct smear were performed in the referral hospital due to limited resources in the community health center. Cerebral schistosomiasis diagnosis was verified based on brain tissue biopsy, stool examination, and CT scan results. Besides the craniotomy, the doctor admitted the patient with a combination of steroids and PZQ at a dosage of 60 mg/kg single dose. She was released after 14 days in satisfactory overall health. The follow-up CT scan revealed improvement, corroborated by the patient's clinical recovery. The patient exhibited a highly favorable response to PZQ therapy.

## 3. Discussion

Over 200 million individuals globally are believed to be infected with schistosomiasis due to exposure to contaminants [4]. Cerebral schistosomiasis is an uncommon ailment in which *S. japonicum* frequently precipitates cerebral lesions; in contrast, spinal cord lesions are frequently caused by *S. mansoni* and *S. haematobium* [25–27]. The distribution of the disease is concentrated in tropical and subtropical regions, particularly within socioeconomically disadvantaged communities deficient in clean water and sanitation infrastructure [28]. The infection in humans arises when cercariae, the larval form of the parasite discharged by freshwater snails, penetrate the skin during exposure to water polluted with urine or feces from schistosomiasis patients. The cercaria larva penetrates the human bloodstream [2, 16]. Following initial infection, the parasites relocate to the inferior mesenteric arteries within days, commencing oviposition within 6 weeks, and sustaining this reproductive activity for their estimated lifespan of three to 5 years. The eggs traverse the blood vessel lumen and intestinal mucosa, ultimately leading to the liberation of miracidia within fecal matter, thereby completing the parasite's life cycle [6, 29]. Schistosomiasis in Indonesia is geographically restricted to Central Sulawesi Province, notably in the Napu and Bada Plateaus of Poso Regency and the Lindu Plateau within Sigi Regency. Currently, *S. japonicum* remains endemic in these three remote regions. The infection is transmitted through *S. japonicum*, with *Oncomelania hupensis lindoensis* snails serving as the intermediate host [30].

Various factors underlie the development of cerebral schistosomiasis, among which the inflammatory reaction and the migration of schistosome eggs or mature worms into the CNS are paramount. The first route, egg embolization, refers to schistosome eggs entering the brain through venous shunts, especially in individuals with hepatic and pulmonary hypertension. This is a frequently traveled route for eggs to reach the CNS, mainly from the liver and lungs [20]. Secondly, in the case of aberrant migration, adult worms can migrate to the vertebral venous plexus, also known as the Batson plexus, which is a network of veins that do not have valves. This lets the worms move and lay eggs directly into the brain, forming granulomatous lesions. The third pathway involves the activation of a granulomatous inflammatory response in the brain due to the presence of schistosome eggs. This reaction entails creating granulomas, clusters of immune cells that strive to encase and eradicate the foreign eggs. This process has the potential to cause substantial harm to the tissues and result in neurological problems. If the brain is affected, it often results in seizures. If the spinal cord is involved, it can cause spinal cord compression and paralysis. The final aspect is the robust immunological response, which can result in substantial tissue damage in reaction to schistosome eggs. The eggs exhibit a high level of immunogenicity, stimulating immunological responses at the administration site and throughout the body. This heightened immune response contributes to the formation of granulomas and the accompanying neurological symptoms [23, 31]. In this present case, the route of the cerebral schistosomiasis development was the first route and the third pathway because histopathological analysis only found *S. japonicum* eggs, and no adult worms were found in the brain tissue.

The standard parasitological methods are the most traditional among the several diagnostic procedures. The diagnostic approaches employed include direct microscopic identification of miracidia hatching and eggs in fecal samples for intestinal schistosomiasis or urine samples for urogenital schistosomiasis. Despite its limitations, microscopic analysis of urine and feces is still the gold standard for diagnosing schistosomiasis. Conventional methods were labor-intensive and exhibited low sensitivity and specificity [32]. When eggs are not detected, serological testing can serve as a supportive diagnostic method; however, its reliability is limited due to the potential for high false-positive rates, making it less suitable for definitive diagnosis. In cases where the clinical presentation is unusual and standard laboratory findings are inconclusive, a biopsy may be beneficial. This procedure can reveal schistosome eggs encased within granulomatous tissue, providing clearer diagnostic confirmation [33]. In a prior investigation, cerebral schistosomiasis due to *S. japonicum* was diagnosed in a 3-year-old girl using a combination of advanced imaging modalities, histopathology, and metagenomic next-generation sequencing (mNGS), which confirmed the diagnosis despite negative serological tests [34]. Our case report did not utilize serological testing due to the limited availability of diagnostic facilities in the referral hospital in our region. Therefore, the diagnosis was primarily established based on histopathological findings from brain tissue biopsy, stool examination, and radiological imaging.

Various new ways were developed to regularly measure the host immune response by detecting antibodies to crude or purified antigens from adult worms and eggs. The goal of immunological tests is to detect the presence of schistosomal antigens or antischistosomal immunoglobulins in biological fluids, including serum, urine, or sputum. Incorporating immunological assays alongside standard diagnostic procedures can enhance detection, especially when conventional methods fail to identify infections in patients with low intensity. The development of serological tests has led to the availability of efficient and fast testing options that can be used in both public and facility-based settings in areas with high disease prevalence. Limitations of serological testing encompass the lack of discrimination between current and past infections, as well as the possibility of cross-reactivity among antigens, and the fact that the reagents and trained personnel necessary to conduct the examinations are not available in all laboratory facilities, particularly in remote areas [32].

Primary involvement of the CNS can occur in either the brain or the spinal cord. Prominent early symptoms identified encompass headache, cognitive dysfunction, generalized tonic–clonic seizures, localized neurological impairments, impaired memory retention, and delayed cognitive responses, resembling the clinical profile of cerebral schistosomiasis [20, 25, 35]. The constellation of acute neurological deficits, significant eosinophilia, and numerous cerebral infarcts supports the hypothesis that eosinophil activation in response to larval migration induces vasculitis and thrombosis in small cerebral vessels [36]. Histological examination usually shows the development of granulomas surrounding eggs deep inside the afflicted tissue, which aligns with this patient's histopathological result. MRI is often considered the most effective imaging technique for diagnosing cerebral schistosomiasis [23]. The patient underwent imaging mainly via a CT scan due to financial limitations, which revealed a hypodense zone in the left parieto-occipital area accompanied by perifocal edema, aligning with typical CT scan findings of cerebral schistosomiasis [27].

Based on these case reports, after the partial craniotomy and administering PZQ and steroid combinations for 2 weeks, the patient showed clinical recovery. Surgery for cerebral schistosomiasis is primarily indicated in two situations: (1) when a definitive diagnosis is needed through biopsy and (2) in cases with severe neurological symptoms and CSF flow obstruction [26]. The surgical treatment of cerebral schistosomiasis generally produces positive outcomes when paired with suitable medicinal therapy. Most patients have significant neurological enhancement between 1 and 6 months following surgery. Cerebral schistosomiasis instances typically yield superior outcomes relative to spinal situations. Thorough excision of lesions frequently alleviates symptoms [21]. The pharmacotherapy of cerebral schistosomiasis necessitates a meticulous strategy that integrates both antiparasitic and steroid pharmacotherapy. Steroid treatment goals are to mitigate inflammation and granuloma size and assist in managing inflammatory reactions that may arise following the death of adult worms [37]. PZQ employs many ways to attack schistosomes. However, its precise chemical action is not entirely elucidated. The PZQ disturbs calcium ion homeostasis in parasitic worms by antagonizing voltage-gated calcium channels, inducing unregulated calcium influx, and resulting in muscular contraction and paralysis. PZQ also inflicts considerable harm to the parasite's tegument by modifying the surface membrane architecture, revealing concealed parasitic antigens, and rendering the worm susceptible to host immunological reactions [38–40].

## 4. Conclusion

This case highlights the diagnostic challenges of cerebral schistosomiasis in a remote, resource-limited setting in Indonesia. Despite the absence of serological testing, a definitive diagnosis was successfully established through radiological imaging, stool microscopic examination, and brain tissue biopsy (histopathological analysis) which revealed *Schistosoma* eggs surrounded by granulomatous inflammation. The patient presented with space-occupying brain lesions and neurological symptoms, but without hepatic involvement, making the diagnosis less straightforward. This case underscores the importance of considering cerebral schistosomiasis in the differential diagnosis of cerebral lesions in endemic areas. Diagnosis of cerebral schistosomiasis based on a detailed social occupational history correlated with radiological imaging, stool microscopic examination, and brain tissue biopsy (histopathological analysis) is essential when other diagnostic tools (serological testing) are unavailable [41].

## Figures and Tables

**Table 1:** Summary of laboratory examination.

Variable	Reference range	Results
Leukocyte (10^3^/μL)	4–10	9.45
Neutrophil (10^3^/μL)	2–7	4.74
Lymphocyte (10^3^/μL)	0.8–4	2.44
Monocyte (10^3^/μL)	0.12–1.2	0.46
Eosinophil (10^3^/μL)	0.02–0.5	1.74 (high)
Basophil (10^3^/μL)	0–0.1	0.07
Erythrocyte (10^6^/μL)	3.5–5	4.76
Hemoglobin (g/dL)	11–15	12.3
Hematocrit (%)	37–47	37.3
Platelet (10^3^/μL)	150–450	358
Procalcitonin (%)	0.108–0.282	0.317 (high)

## Data Availability

The data used to support the study are included within the article.
